# Supplementary material for: Aerobic exercise interventions to address impaired quality of life in patients with pituitary tumors
Source: PLoS One. 2023 Dec 15;18(12):e0295907. doi: 10.1371/journal.pone.0295907 (PMC10723697; doi:10.1371/journal.pone.0295907)
Supplement: Attachment — Submitted filename: response to reviewers.docx [file pone.0295907.s001.docx]

**Additional Editor Comments:**

Based on the reviewers' suggestions, the paper needs major revision.  The reviewers' comments can be found below.

**Response:** Thank you for the opportunity to revise our manuscript. Please find below our responses to each reviewers’ suggestions.

Reviewer #1: Reviewer’s comments
Manuscript number: PONE-D-23-27034
Title: Aerobic exercise interventions to address impaired quality of life in patients with pituitary tumors

Summary
In this review article, the authors summarize previously reported literatures on aerobic exercise interventions in patients with pituitary tumors. This review article looks at what we already know about using exercise to help pituitary tumor patients. It also talks about things we should study in the future, like designing exercise programs based on the patient's hormones and using better ways to see if the exercise is working.

Overall, it's a well-written paper. However, there are some areas that need improvement, such as:

1. More detailed explanations are needed in the 'Method' section, particularly regarding the inclusion and exclusion criteria. Including figures or charts that show the total number of papers searched and the number excluded would be helpful.

**Response:** We have added commentary into the methods on our inclusion and exclusion criteria and also created a flow diagram (figure 1) demonstrating our search strategy for the literature review.

2. Summarizing relevant papers related to the topic of this review article in a table would be very useful.

**Response:** We have summarized the relevant papers in newly created Table 1.

3. Providing more detailed descriptions of areas requiring future research in the conclusion section would be beneficial."

**Response:** We have added additional commentary on specific areas where future research is needed in the conclusion section.

Overall impression

I think that this manuscript contains valuable information worthy of publication in PLOS ONE, after major revision has been made. I would recommend resubmission of the manuscript including considerable revisions.
Thank you very much.

**Response:** We appreciate the reviewer’s effort and time to evaluate our manuscript. We hope our edits suffice.

Reviewer #2: The authors have undertaken a literature review on the really interesting topic of fatigue and exercise interventions after pituitary surgery. This is an underrepresented topic in the literature, but a very important quality of life issue. The authors should be commended on undertaking this review and highlighting the problem.
The manuscript is on the whole very well written and a very useful addition to current literature.
Some areas could be improved:
1. The methodology is sparse, only one database was searched. Inclusion and exclusion criteria could be better defined. I accept that the authors were not intending to undertake a systematic review, but the methodology could be much more robust. A table of the papers included would be useful.

**Response:** We have provided a more detailed explanation of our inclusion/exclusion criteria in the Methods section and created a flow diagram, outlining our search strategy (figure 1). Additionally, we have provided a review of the papers in Table 1.

2. line 77, validated health related questionnaires - state which
line 79 typo scores fatigue

**Response:** We have clarified the health related questionaries in line 77 and provided relevant citations. We have fixed this typo in line 79.

3. Results section impaired quality of life - this section includes exercise outcomes which should be in the subsequent section. It also lacks detail and is difficult to follow. There should be a better description of the results of the Qol outcome measures in the studies

**Response:** We have edited this section to clarify that the exercises outcomes related to the study by Zhao et al, was an observational study rather than a deliberate interventional study, showing that patients who self reported increased exercise activity after surgery also had lower levels of fatigue. This study differs from those we present in the subsequent section which are specifically describing prospective interventions via exercise regimens and their outcomes. Additionally, we have provided additional details on the methodologies and results on the QoL outcome measures with relevant citations for all of the studies.

4. Line 225 - this section of the discussion should be sub headed conclusion

**Response:** We have edited this paragraph to be the conclusion section.
